# Purtscher-Like Retinopathy Associated with Atypical Hemolytic Uremic Syndrome

**DOI:** 10.4274/tjo.66502

**Published:** 2017-12-25

**Authors:** Melih Ustaoğlu, Feyza Önder, Nilgün Solmaz, Savaş Öztürk, Mesut Ayer

**Affiliations:** 1 University of Health Sciences, Şişli Hamidiye Etfal Training and Research Hospital, Ophthalmology Clinic, İstanbul, Turkey; 2 University of Health Sciences, Haseki Training and Research Hospital, Ophthalmology Clinic, İstanbul, Turkey; 3 University of Health Sciences, Haseki Training and Research Hospital, Nephrology Clinic, İstanbul, Turkey; 4 University of Health Sciences, Haseki Training and Research Hospital, Hematology Clinic, İstanbul, Turkey

**Keywords:** Atypical hemolytic uremic syndrome, Purtscher retinopathy, Purtscher-like retinopathy, thrombotic microangiopathy, eculizumab

## Abstract

A 25-year-old woman presented with acute bilateral blurred vision and history of headache, dizziness, and syncope for three days. Her visual acuity was 20/60 in both eyes. Fundoscopy revealed multiple bilateral peripapillary yellow-white patches like cotton wool spots, intraretinal hemorrhages and macular edema. The patient was diagnosed with Purtscher-like retinopathy based on the retinal findings and lack of trauma history. She was urgently admitted to the nephrology clinic due to thrombotic microangiopathy findings (hemoglobinemia, thrombocytopenia, and acute renal failure). After excluding thrombotic microangiopathy, the patient was diagnosed with atypical hemolytic uremic syndrome (aHUS) with the clinical and laboratory findings. Eculizumab treatment was added to hemodialysis and plasmapheresis therapy. Three months after starting treatment, retinal lesions regressed and visual acuity increased to 20/20 in both eyes. To the best of our knowledge, this is the first reported case of Purtscher-like retinopathy associated with aHUS.

## INTRODUCTION

Purtscher’s retinopathy is a rare retinal disorder characterized by acute visual loss and retinal findings such as cotton-wool spots, intraretinal hemorrhages and retinal whitening following head or chest trauma.^1^ When the etiology is not a trauma, the disease is called Purtscher-like retinopathy. Numerous conditions such as acute pancreatitis, connective tissue disorders, autoimmune diseases, pregnancy-related diseases, and thrombotic microangiopathic diseases can cause Purtscher-like retinopathy.^2^

Atypical hemolytic uremic syndrome (aHUS) is a very rare life-threatening disease. It is characterized by the triad of microangiopathic hemolytic anemia, thrombocytopenia, and renal failure.3 It is differentiated from hemolytic uremic syndrome (HUS) by the absence of diarrhea and Shiga toxin-induced infection.4 The main pathology of aHUS is dysregulation of the complement system, leading to vascular endothelial damage and complement aggregations.

## CASE REPORT

**Figure 1:**
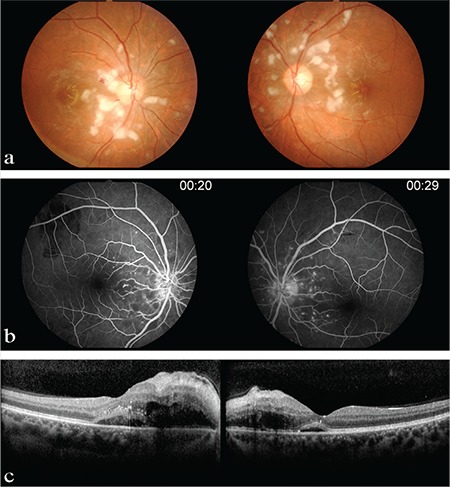
Initial fundus photography, fluorescein angiography and optical coherence tomography findings: (a) Bilateral multiple peripapillary yellow-white patches, flame-shaped intraretinal hemorrhages, and macular edema (b) Bilateral peripapillary hyperfluorescent spots (c) Serous macular detachment at optical coherence tomography in both eyes

A 25-year-old woman presented with acute bilateral blurred vision and history of headache, dizziness, and syncope for three days. Her medical history was unremarkable except for migraine attacks since childhood. On ophthalmoscopic examination, her best-corrected visual acuity was 20/60 in both eyes. Anterior segment examination was unremarkable and intraocular pressures were within normal limits. Fundoscopy revealed bilateral multiple peripapillary yellow-white patches like cotton-wool spots, flame-shaped intraretinal hemorrhages, and macular edema (Figure 1a).

After urgent ophthalmoscopic examination, an internal medicine specialist was consulted due to accompanying symptoms. Blood pressure was 140/90 mmHg and body temperature was 37.2 °C in her systemic evaluation. Laboratory tests of the patient revealed hemoglobinemia (9.2 g/dL), thrombocytopenia (66,000/mL), increased levels of blood lactate dehydrogenase (1687 U/L), indirect bilirubin (1.69 mg/dL), creatinine (4.8 mg/dL), C-reactive protein (28 mg/dL), and blood urea nitrogen (162 mg/dL), and decreased blood haptoglobin levels (1.9 mg/dL). Prothrombin time (PT), activated partial thromboplastin time (aPTT), and fibrinogen levels were within normal limits.

The patient was hospitalized in the nephrology clinic due to accompanying acute kidney failure and she was scheduled for hemodialysis and plasmapheresis. The day after admission, we performed optical coherence tomography (OCT) and fluorescein angiography were performed. Fluorescein angiography showed bilateral peripapillary hyperfluorescent spots (Figure 1b). OCT revealed serous macular detachment in both eyes (Figure 1c). Due to the corresponding retinal findings and lack of trauma history, the patient was diagnosed with Purtscher-like retinopathy and the treatment of underlying systemic pathology was recommended.

In the nephrology clinic, a blood smear test, abdominal ultrasonography, and ADAMTS13 tests were performed for the differential diagnosis of acute kidney failure. The blood smear test showed schistocytes and erythrocyte fragmentation, and ADAMTS13 test was negative. Abdominal ultrasonography revealed bilateral grade 2 renal parenchymal hyperechogenicity with normal kidney sizes. After the systemic examinations and laboratory tests, our patient was evaluated as having thrombotic microangiopathy due to hemoglobinemia, thrombocytopenia, and acute renal failure. In the differential diagnosis of thrombotic microangiopathy, HUS was eliminated due to the absence of Shiga toxin-induced infection and bloody diarrhea; disseminated intravascular coagulation (DIC) was excluded based on normal PT, aPTT, and fibrinogen levels; and thrombotic thrombocytopenic purpura (TTP) was excluded due to the negative ADAMTS13 test; based on the laboratory and clinical findings, the patient was diagnosed with aHUS. Eculizumab, which is a humanized monoclonal antibody that blocks complement activity by cleavage of the complement protein C5, was added to the hemodialysis and plasmapheresis treatment. The eculizumab treatment was initiated at 900 mg weekly for the first four weeks, and then continued at 900 mg every three weeks.

**Figure 2:**
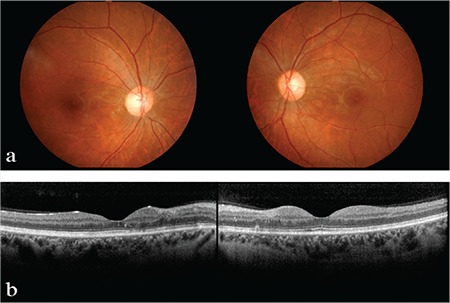
Fundus photography and optical coherence tomography findings in the third month of follow-up: (a) Total resolution of the retinal findings (b) Complete regression of subretinal fluid demonstrated by optical coherence tomography

Three months after starting treatment, her visual acuity increased to 20/20 in both eyes. Fundoscopy showed improvement of the retinal lesions (Figure 2a) and OCT revealed total regression of the macular edema (Figure 2b). The patient was followed for two years under treatment with eculizumab and no recurrence was observed.

## DISCUSSION

Purtscher-like retinopathy is a very rare retinal disorder with an incidence rate of 0.24 patients per million per year.^1^ The most encountered signs of this retinopathy are cotton-wool spots, retinal hemorrhages, Purtscher flecken, pseudo-cherry red spot, and macular edema, respectively.^2^ The generally accepted pathophysiology of Purtscher-like retinopathy is vascular endothelial damage and arteriolar precapillary occlusion by emboli of leucocytes, fibrin, fat, and complement aggregates. This retinopathy is mostly seen with acute pancreatitis, renal failure, autoimmune diseases, and thrombotic microangiopathies such as TTP, HUS, and DIC.

aHUS is a thrombotic microangiopathy caused by mutations of factor H, factor I, factor B, membrane cofactor protein, C3 convertase component, and thrombomodulin gene.^4^ These abnormalities lead to dysregulation of the complement alternative pathway, which causes thickening of arterioles and capillaries, endothelial detachment, subendothelial accumulation of proteins, cell debris, and fibrin-platelet thrombi obstruction.^3^ This pathogenesis of aHUS leads to systemic multi-organ involvement, and there are only a few reports showing the ocular involvement of aHUS in literature.^5,6,7^ Zheng et al.^5^ reported a case with recurrent ocular involvement which was consistent with central retinal vein occlusion/venous stasis retinopathy in the first attack, and inferior rectus paralysis in the second attack and was treated with steroids. Larakeb et al.6 reported a case of vitreous bleeding due to aHUS, and their patient improved with plasma exchange therapy after four weeks. David et al.^7^ described a patient with aHUS and serous retinal detachment who was treated with hemodialysis, plasmapheresis, and eculizumab. That case shares many similarities with our patient: same age, female sex, similar ocular findings, and successful response to eculizumab treatment. However, their patient had a lesser extent of retinal yellow-white patches compared to our patient. To the best of our knowledge, our patient is the first reported case of Purtscher-like retinopathy associated with aHUS.

Purtscher-like retinopathy is a very rare retinal disorder which is commonly caused by thrombotic microangiopathic diseases such as HUS and aHUS. These diseases are severe, life-threatening diseases and mostly occur in childhood or early adulthood. Therefore, performing a detailed fundus examination in every patient, especially in the pediatric age group, is crucial for recognizing these retinopathies which are caused by underlying life-threatening diseases.
